# Design optimization of health apps for rural older adults: enhancing usability and health effects based on Kano model and SEM method

**DOI:** 10.3389/fpubh.2026.1810287

**Published:** 2026-07-03

**Authors:** Hongling Jiang, Hong Li, Xuan Li, Qiang Du

**Affiliations:** 1 Guangzhou Huashang College, Guangzhou, China; 2 Tianjin Tianshi College, Tianjin, China

**Keywords:** age-friendly design, health apps, Kano model, rural older adults, structural equation model (SEM), user satisfaction

## Abstract

**Introduction:**

With the intensification of population aging in rural China, older adults face multiple challenges in health management, particularly barriers to using health apps, such as operational complexity, unfriendly interfaces, and insufficient contextual adaptation. This study aimed to optimize the design of health apps for rural older adults by identifying user requirements and examining how design optimization affects health management outcomes.

**Methods:**

This study employed an integrated Kano model and structural equation modeling (SEM) approach. First, the needs of rural older adults were investigated, and a set of health app design elements was constructed. Then, the Kano model was used to classify these design elements into Basic Requirements, Expected Requirements, and Attractive Requirements. Finally, SEM was used to analyze the relationships among design requirements, User Satisfaction, Intention to Use, and User Loyalty.

**Results:**

The findings showed that meeting Basic Requirements and Expected Requirements significantly improved User Satisfaction, Intention to Use, and User Loyalty among rural older users. Attractive Requirements further enhanced emotional experience and long-term loyalty through innovative features such as dialect support and social interaction. User Satisfaction was also found to play an important mediating role in strengthening users’ intention and loyalty.

**Discussion:**

These findings suggest that the design optimization of health apps can effectively reduce usage barriers for rural older users and improve their health management outcomes. This study provides theoretical support for the age-friendly design of health apps and practical guidance for promoting the popularization and application of digital health management tools among rural older adults.

## 1 Introduction

### 1.1 Research background

China’s population aging is accelerating, and this trend is particularly evident in rural areas. According to the Seventh National Population Census, China had 264.02 million people aged 60 and above in 2020, accounting for 18.70% of the total population, while 190.64 million people were aged 65 and above, accounting for 13.50% of the total population (1). More recent national statistics further show that China’s population aged 60 and above reached nearly 297 million in 2023, accounting for 21.1% of the total population, indicating that population aging has become a major demographic and social governance issue (2). Compared with older adults in urban areas, rural older adults face more complex challenges, including limited medical resources, inconvenient transportation, lower digital literacy, and weaker social support systems. These factors place them at a disadvantage in accessing health management and healthcare services (3). Therefore, in the Chinese context, optimizing health apps for rural older users is not only a technical issue of improving app usability, but also an important response to population aging, digital inclusion, and the equalization of primary healthcare services. As rural aging deepens and digital health services expand, whether rural older users can effectively access and use health apps has become closely related to health equity and the modernization of grassroots health governance. Health apps can serve as a digital bridge linking rural older users, family support, village doctors, community health services, and local medical resources. Therefore, age-friendly and context-sensitive app design is essential for reducing the digital divide, improving daily health management, and enhancing the accessibility of healthcare services for rural older adults (4).

With the continuous development of smart technologies and health management tools, health apps have gradually become important tools for improving health management among older adults (5). In rural areas, health apps can provide services such as telemedicine, health monitoring, medication reminders, health knowledge support, and psychological counseling, thereby supplementing traditional healthcare services and supporting daily self-management (6). However, despite their application potential, the actual use of health apps among rural older users remains limited. Many rural older users still face barriers such as complex operation procedures, unfriendly interfaces, insufficient voice support, limited digital literacy, privacy concerns, and a mismatch between app functions and daily health management requirements (7). Prior research on rural patients with chronic diseases has also shown that rural users face specific barriers when using mobile health tools, including limited technological support, insufficient localized content, and difficulties in integrating app-based services into daily contextual requirements (8). Therefore, the key issue is not only whether health apps can provide health-related services, but also whether their design can effectively respond to the cognitive abilities, usage habits, and contextual needs of rural older users. Exploring the barriers to health app use in rural areas and identifying how design optimization can improve rural older users’ experience has therefore become an important research issue. This study focuses on the design optimization of health apps for rural older users, with particular attention to how different types of design requirements influence User Satisfaction, intention to use, and User Loyalty.

### 1.2 Literature review and research gap

In recent years, research on health app design for older adults has mainly focused on interface usability, accessibility, and technology acceptance. From the perspective of interface and interaction design, existing studies have shown that font size, icon clarity, color contrast, navigation simplicity, and accessibility directly affect older users’ usability experience and adoption behavior (9, 10). Previous research has further indicated that simplified interfaces, reduced operational complexity, and age-friendly interaction strategies play an important role in improving older adults’ mobile health app experience (11). Interface-level studies have also shown that visual hierarchy, information density, and visual search efficiency significantly influence older adults’ app use experience (12). From the perspective of technology acceptance, the Technology Acceptance Model and its extended models have been widely used to explain users’ behavioral intentions toward digital technologies and health app use (13–15). Empirical studies on mobile health use among older adults have further shown that health literacy, digital self-efficacy, social support, perceived barriers, and privacy concerns are closely associated with health app acceptance and continued use (16). These studies provide an important foundation for understanding older adults’ health app use behavior. However, most studies explain health app use mainly from the perspectives of interface usability and technology acceptance, and have not sufficiently distinguished the roles of different app functions across levels of user requirements, namely which functions constitute basic acceptance conditions, which provide expected functional support, and which enhance emotional experience. Therefore, how different levels of design requirements jointly influence User Satisfaction and continued use behavior remains to be further clarified.

In China, health apps have gradually been integrated into the digital health service system, including online consultation, appointment registration, medication purchasing, health monitoring, and health knowledge dissemination (17). However, the diffusion and actual use effects of health apps still vary across regions and user groups. Compared with urban users, rural older users face more constraints in digital literacy, network access, linkage with local healthcare services, and app adaptability (18). This uneven diffusion indicates that health app optimization in the Chinese context should not be understood only as a general issue of interface improvement, but should be analyzed in relation to regional healthcare accessibility and the specific requirements of rural older users.

From the perspective of requirement analysis, health app optimization requires a clearer understanding of different levels of user requirements. Existing studies on mobile health app quality evaluation have identified security, privacy protection, reliability, usability, and information transparency as important evaluation criteria (19). For older adults, these criteria are closely related to trust building, health data security, medical consultation, medication management, and long-term health management. However, different requirements do not affect User Satisfaction in the same way. The Kano model has been applied to health app evaluation and healthcare requirement analysis because of its ability to classify user requirements according to their differentiated effects on satisfaction. Malinka et al. used the Kano model to prioritize quality principles for health apps, showing that different app quality attributes contribute differently to User Satisfaction (20). Yan et al. applied the Kano model to analyze the self-management needs of middle-aged and older patients with chronic kidney disease, revealing heterogeneity in user requirements across different groups (21). For health apps, some functions constitute basic conditions for user acceptance, such as privacy protection, interface readability, and information reliability; some functions directly enhance perceived usefulness, such as voice assistance, medical consultation, health monitoring, and medication reminders; and other functions, such as dialect support, family health sharing, social interaction, and localized content, can enhance emotional experience and increase users’ willingness to continue using the app. In this study, the must-be, one-dimensional, and attractive attributes in the Kano model correspond to Basic Requirements, Expected Requirements, and Attractive Requirements, respectively. Therefore, the Kano model is suitable for identifying different levels of requirements in health app design.

For rural older users, health app use is not merely an individual technology-use behavior, but is deeply shaped by healthcare service conditions and social support environments. Existing studies have shown that rural older adults in China still face multiple constraints in digital technology use, social support, geographic accessibility, and access to health management services (22, 23). In addition, studies on rural patients with chronic diseases have shown that rural users face specific barriers when using mobile health tools, including limited technological support, insufficient localized content, and difficulties in integrating app-based services into daily contextual requirements (24). Compared with general older users or urban older users, rural older users face more prominent constraints, including limited local medical resources, lower digital literacy, stronger needs for dialect-based communication, and insufficient linkage between digital tools and village-level healthcare services. Therefore, health apps for rural older users should not be understood merely as individual self-management tools, but also as digital interfaces connecting family support, village doctors, community health services, and local medical resources. This contextual difference indicates that findings derived from general older users need contextual adaptation before they can be effectively applied to rural older adults.

However, relying solely on the Kano model is still insufficient to explain the behavioral mechanisms underlying health app use. The Kano model can identify which design requirements are basic, expected, or attractive, but it cannot further test how these different types of requirements influence User Satisfaction, intention to use, and User Loyalty. In technology acceptance and user behavior research, structural equation modeling has been widely used to examine relationships among latent variables, such as perceived usefulness, satisfaction, behavioral intention, and continued use. This method is particularly suitable for testing whether theoretical constructs can explain behavioral outcomes through statistical paths. Existing SEM studies emphasize the importance of standardized parameter estimation when validating relationships among latent constructs (25). In addition, model fit evaluation is also essential for assessing whether the proposed structural model adequately represents the observed data (26). Therefore, this study integrates the Kano model with SEM to connect requirement classification with behavioral mechanism testing. The Kano model first classifies rural older users’ health app requirements into different levels, while SEM further examines how these requirement levels influence User Satisfaction and how satisfaction affects intention to use and User Loyalty. This two-stage analytical logic enables the study to move from design element identification to behavioral mechanism verification.

Based on the above literature review, three main research gaps remain. First, most studies on health app design focus on general older adults or urban older adults, while insufficient attention has been paid to the specific usage contexts of rural older users, including limited medical resources, differences in digital capability, dialect communication requirements, and linkage with local healthcare services. Second, existing studies often explain health app use problems through isolated interface elements or operational barriers, and lack a systematic design framework that integrates basic usability, functional support, emotional experience, and localized services. Third, existing applications of the Kano model mainly focus on requirement classification and priority judgment, and rarely combine Kano-based requirement classification with SEM to analyze how different types of requirements influence satisfaction, intention to use, and User Loyalty. Therefore, the core research question of this study is not only which health app functions rural older users need, but also how different levels of design requirements affect their satisfaction, and how satisfaction further influences intention to use and User Loyalty.

The contributions of this study are mainly reflected in three aspects. First, this study shifts the research object of health app design from general older adults to rural older users, responding to practical issues such as rural aging, digital inclusion, and insufficient access to primary healthcare services. Second, this study constructs an integrated analytical framework combining the Kano model and SEM, linking user requirement classification with behavioral path testing, and explaining how Basic Requirements, Expected Requirements, and Attractive Requirements influence User Satisfaction and subsequent behavioral outcomes. Third, from the perspective of design studies, this study proposes optimization priorities for health apps in rural contexts, providing design evidence for age-friendly interaction design, localized health content organization, privacy and trust mechanism construction, and linkage with primary healthcare services.

## 2 Methods

Based on this context, this study employed an integrated Kano model and SEM approach to identify and analyze the key factors influencing design optimization and how these factors affect the health management outcomes of the older adults by enhancing the user experience. In this study, the Kano model was not used to directly replace the latent variable structure of SEM. Rather, it served as a preliminary requirement classification method to identify the dominant quality attributes of specific health app design elements. SEM was then used to examine the structural relationships among the aggregated requirement constructs, User Satisfaction, Intention to Use, and User Loyalty. Therefore, the integration of Kano and SEM was designed as a two-stage analytical process: the Kano model was used for requirement classification and prioritization, while SEM was used for mechanism verification and path analysis.

### 2.1 Research process

To systematically investigate the impact of health app design on older users in rural areas, this study follows the following steps:

**The first step was to build a pool of design elements based on literature review and preliminary research.**

First, through a systematic review of domestic and international literature on the design of health apps for the older adults, age-friendly design, the digital divide in rural areas, and health information behavior, and in combination with preliminary interviews with rural older users, healthcare workers, and community workers, fully identify design elements that may affect the user experience and acceptance of health apps for the rural older adults. These elements cover multiple dimensions, including interface design (such as font size, icon clarity, color contrast), interaction logic (such as navigation complexity, operation steps, error prevention), functional Settings (such as highlighting core functions, functional redundancy, information architecture), and content presentation (such as language accessibility, information credibility, multimedia applications). Through this process, a preliminary pool of design elements is constructed, providing a systematic framework for subsequent analysis.

**The second step was to use the Kano model to identify key design elements and their attributes.**

Based on the pool of design elements constructed in the first step, a standardized Kano questionnaire was designed. By setting a pair of positive and negative questions for each design element, the questionnaire asked rural older adult respondents to choose their satisfaction levels based on their feelings. By analyzing the Kano model, each design element was precisely classified as: essential element, expected element, attractive element, undifferentiated element, and inverse element (27). This analysis helped identify the most critical design elements, providing strong support for the subsequent SEM analysis.

In this study, Kano categories were not directly treated as SEM latent variables. The Kano model was first used to classify the dominant quality attributes of health app design elements. Based on the theoretical logic of the Kano model, the design elements classified as must-be, one-dimensional, and attractive attributes were aggregated into three requirement-related constructs: Basic Requirements, Expected Requirements, and Attractive Requirements. These constructs were then tested in SEM for their effects on User Satisfaction, Intention to Use, and User Loyalty. All SEM items were measured using a five-point Likert scale and standardized before model estimation. No additional subjective weighting was applied; the relative effects were estimated using standardized path coefficients.

**The third step was to construct and validate the structural equation model (SEM) for the impact mechanism.**

Building on the key design elements identified by the Kano model, this study further constructs an initial conceptual model. The model assumes that the core design constructs (from the key design elements screened out by the Kano model, particularly the expectancy and charm elements) directly affect the user’s “Intention to Use” and perceived “obstacles to use.” “Usage barriers” include dimensions such as operational complexity, insufficient interface friendliness, and poor functional adaptation, which directly affect users’ “continuous usage behavior” and ultimately ‘health management outcomes’ such as the frequency of self-health monitoring, access to health knowledge, and improvement of medical compliance. In addition, user characteristics, such as age, educational attainment, digital literacy, and health status, as control or moderating variables may influence the aforementioned pathway relationships.

To achieve this, a structured questionnaire was designed to cover all potential variables and their corresponding observed variables (28). The questionnaire items were carefully designed to ensure their reliability and validity. Subsequently, a large-scale questionnaire survey was conducted on a representative sample of rural older adults, and SEM analysis was performed using the statistical software AMOS.

In summary, this study combined the Kano model and SEM through a sequential analytical logic. The Kano model was responsible for identifying and classifying micro-level design elements according to rural older users’ differentiated requirement attributes, while SEM was responsible for verifying the macro-level causal paths among requirement constructs, User Satisfaction, Intention to Use, and User Loyalty. The integration of the two methods enabled this study to move from descriptive requirement classification to explanatory mechanism testing, thereby providing both design prioritization and empirical validation for health app optimization targeting rural older users.

### 2.2 Build a set of user requirements

In the stage of constructing the user requirements set in this study, rural older adults were selected as the research subjects. By using a prepared interview framework and purposivel sampling, in-depth interviews were conducted with rural older adults with health management requirements to ensure that the core requirements of users were truly identified (29). The entire interview cycle lasted for 7 days and was conducted in both face-to-face and telephone formats. A total of 35 interviewees, aged between 60 and 80, were interviewed, including 19 females and 16 males. The occupations of the respondents mainly included farmers, retired teachers and rural medical service workers. Some of the respondents have long relied on traditional medical services, while others have tried using smart devices or apps for health management. During the interviews, the research team encouraged respondents to think as broadly as possible within the framework questions and talk about their health requirements and their perceptions of technology (30). The interview questions revolved around health management requirements, awareness of apps, obstacles to using smart devices, and the need for health information (31). Through the interviews, the research team collected rich data, collated all respondents’ feedback, and refined and integrated the collected user requirements through group discussions and requirement extraction (32). Ultimately, five categories of functional requirements for rural older adults were obtained to construct the user requirements set (as shown in Table 1).

**Table 1:** Elements and descriptions of age-friendly user requirements for healthcare apps.

Classification	Serial number	Elements	Explanation
Interface and interaction design	1	Interface Friendly design	Large fonts, large ICONS, clear layout, high color recognition, and reduced complexity
	2	Real-time feedback mechanism	When the operation succeeds or fails, provide clear voice prompts to reduce operational confusion for the older adults
	3	Reduce advertising	Avoid irrelevant or misleading ads to enhance the user experience
	4	Offline use features	Support browsing basic health knowledge, disease prevention guidelines, etc. in unstable or no network conditions, and provide reminder features
Voice and emergency support	5	Voice assistance features	Provide voice reading, voice search, voice input, and voice reminders
	6	Dialect support	Provide multiple dialect speech recognition capabilities to reduce operational obstacles caused by non-standard Mandarin
	7	Seek help urgently	Automatically send geographical location information to bound emergency contacts (such as family members, village doctors, or nearby medical institutions) via one-click trigger mechanism
	8	Human customer service support	Help seniors solve app usage problems or answer health inquiries in real time via phone or online chat
Health management and services	9	Medical consultation support	Connect local village doctors with remote specialists to support video consultations and online medical advice
	10	Medicine purchase and delivery	Offer online purchase and door-to-door delivery of medicines from local pharmacies
	11	Medical Policy Inquiry	Help seniors quickly understand the latest medical policies, Medicare benefits, reimbursement policies and usage that are relevant to them
	12	Reminder Feature	Reminders include medication reminders, medical reminders, physical examination reminders, and vaccination reminders through various means such as voice announcements, vibrations, and pop-ups
	13	Health monitoring and management	Including features such as step count, dietary guidance, and sleep quality analysis
	14	Infectious disease warnings	Work with local disease control centers to provide up-to-date epidemic information and protection guidelines in a timely manner
	15	Weather forecasts and health advice	Provide real-time weather information and weather forecasts, and health advice based on weather conditions
Health data and privacy protection	16	Health device compatibility	Compatible with common health monitoring devices, it synchronizes health data in real time
	17	Family Health Sharing	Allow children to view the health data of the older adults remotely
	18	Privacy protection	Ensure the security of users’ health data and personal information by providing simple and understandable privacy Settings
Education and Entertainment	19	Online senior classes	Teach the basic operations of smart devices and the common functions of mobile phones through video tutorials and voice explanations
	20	Entertainment Module	Providing content such as opera, music, crosstalk, dance videos, etc., to relieve loneliness and enrich the daily life of the older adults
	21	Social interaction support	Provide a platform for seniors to communicate, reduce loneliness and enhance social interaction
	22	Health knowledge sharing	Disseminate health and wellness knowledge in various forms such as video, text, and voice

### 2.3 Questionnaire design

The questionnaire was developed based on the user requirement set identified in the previous stage. To ensure the completeness and clarity of the measurement process, the questionnaire consisted of three sections: demographic information, the Kano requirement questionnaire, and the SEM measurement questionnaire.

The first section collected respondents’ demographic and background information, including gender, age, current place of residence, experience with smart devices, experience with health apps, and chronic disease status. These variables were used to describe the sample characteristics and to provide contextual information for interpreting rural older users’ health app usage.

The second section was the Kano requirement questionnaire. Based on the 22 age-friendly health app design elements identified in Table 1, each design element was measured through a pair of functional and dysfunctional questions following the standard Kano questionnaire format. Therefore, the Kano questionnaire contained 44 items in total. For example, for the design element “voice assistance function,” the functional question was: “If the health app provides voice reading, voice search, voice input, and voice reminders, how would you feel?” The dysfunctional question was: “If the health app does not provide voice reading, voice search, voice input, and voice reminders, how would you feel?” Respondents answered each item using the standard five-response Kano scale: 1 = “I dislike it,” 2 = “I can tolerate it,” 3 = “I am neutral,” 4 = “It should be that way,” and 5 = “I like it.”

The third section was the SEM measurement questionnaire. Based on the Kano classification results, the design elements classified as must-be, expected, and attractive attributes were further integrated into three requirement dimensions: Basic Requirements, Expected Requirements, and Attractive Requirements. This section was designed to examine the structural relationships among requirement satisfaction, User Satisfaction, Intention to Use, and User Loyalty, rather than to repeat the Kano classification results. In this model, Basic Requirements, Expected Requirements, and Attractive Requirements were treated as antecedent variables affecting User Satisfaction, while User Satisfaction further influenced Intention to Use and User Loyalty. User Satisfaction, Intention to Use, and User Loyalty were measured using items adapted from established scales. Intention to Use was included to capture users’ willingness to continue using the app, while User Loyalty was included to reflect users’ longer-term retention and recommendation tendency. Specifically, User Satisfaction was defined by five observation indicators (US1–US5), including overall satisfaction with the health app, satisfaction with functional usefulness, satisfaction with ease of use, satisfaction with health management support, and satisfaction compared with expectations. Intention to Use was defined by four observation indicators (IU1–IU4), including intention to use the health app in the future, willingness to use the app for daily health management, intention to use the app when health requirements arise, and willingness to increase use of the health app. User Loyalty was defined by three observation indicators (UL1–UL3), including willingness to continue using the health app, willingness to recommend the health app to others, and preference for continuing to use this health app. These items were measured using a five-point Likert scale and were used as observed indicators of the corresponding latent variables in the SEM analysis.

The study selected older adults aged 60 and above in rural areas of Guangdong Province as the research subjects. Convenience sampling was used to collect data from 10 rural communities in Guangdong Province. A total of 370 questionnaires were distributed online. After screening and data cleaning, 339 valid questionnaires were obtained, with a valid response rate of 91.62%, meeting the sample quality requirements of this study.

To further contextualize respondents’ health app usage, the questionnaire also asked participants about the types of health apps they had used or were familiar with. As shown in Table 2, the gender distribution of the respondents was relatively balanced, with a slightly higher proportion of female respondents. In terms of age distribution, the 60–70 age group accounted for the largest proportion. Regarding place of residence, most respondents lived in rural areas, while the rest lived in townships. In terms of smart device experience, 68.73% of the respondents had used smart devices for less than 3 years, while 31.27% had used them for 3 years or more. In terms of chronic disease status, hypertension and diabetes were the most commonly reported chronic conditions. Overall, the sample distribution was generally consistent with the characteristics of rural older users and provided a basis for subsequent analysis.

**Table 2:** Basic information statistics of the survey sample.

Variable	Category	Frequency	Percentage (%)
Gender	Male	166	48.97
	Female	173	51.03
Age	60–65 years old	107	31.56
	65–70 years old	121	35.69
	70–75 years old	78	23.01
	75–80 years old	26	7.67
	Over 80 years old	7	2.06
Current place of residence	Rural	249	73.45
	Townships	90	26.55
Experience with smart devices	Within one year	110	32.45
	One to three years	123	36.28
	Three years and more	106	31.27
Chronic disease status	No	123	36.28
	Hypertension	163	48.08
	Diabetes	46	13.57
	Others	7	2.06

As shown in Table 3, Good Doctor Online had the highest exposure among respondents, with a penetration rate of 38.94%, followed by Baidu Doctor (29.79%), Ping An Good Doctor (27.14%), and DXY Doctor (26.55%). These results indicate that rural older adult respondents had contact with several mainstream health apps. However, app exposure does not necessarily indicate that these apps are fully suitable for rural older users.

**Table 3:** Health apps commonly accessed by rural older adults.

Health app	Response (n)	Response rate (%)	Penetration rate (*n* = 339, %)
DXY Doctor	90	12.68	26.55
114 Health	73	10.28	21.53
Chunyu Doctor	63	8.87	18.58
Baidu Doctor	101	14.23	29.79
Safe and Healthy	68	9.58	20.06
Good Doctor Online	132	18.59	38.94
Ping An Good Doctor	92	12.96	27.14
Ali Health	69	9.72	20.35
Others	22	3.10	6.49
Total	710	100.00	209.44

### 2.4 Kano model analysis and SEM latent variable conversion

To further identify the differentiated requirement attributes of health app design elements, this study first conducted Kano model analysis based on the functional and dysfunctional questionnaire responses. Each design element was classified according to the standard Kano evaluation table into five types: must-be attribute (M), one-dimensional attribute (O), attractive attribute (A), indifferent attribute (I), and reverse attribute (R). The Kano evaluation rules are shown in Table 4.

**Table 4:** Kano model evaluation table.

Functional / dysfunctional	Dislike	Can tolerate	Neutral	It should be that way	Like
Dislike	Q	R	R	R	R
Can tolerate	M	I	I	I	R
Neutral	M	I	I	I	R
It should be that way	M	I	I	I	R
Like	O	A	A	A	Q

A, attractive attribute; O, one-dimensional attribute; M, must-be attribute; I, indifferent attribute; R, reverse attribute; Q, questionable result.

Must-be attributes refer to basic functions that users regard as necessary. If such functions are absent, users are likely to feel dissatisfied, but their presence does not necessarily generate a high level of satisfaction. One-dimensional attributes have a direct and positive relationship with User Satisfaction; the better these functions perform, the higher the level of User Satisfaction. Attractive attributes refer to functions that exceed users’ basic expectations. Their absence may not cause dissatisfaction, but their presence can significantly improve satisfaction. Indifferent attributes have limited influence on User Satisfaction, regardless of whether they are provided.

Based on the Kano classification results, the 22 design elements were further grouped into different requirement categories. The must-be attributes included interface-friendly design, advertising reduction, offline use function, emergency help-seeking, and privacy protection. The one-dimensional attributes included real-time feedback mechanism, voice assistance function, medical consultation support, medicine purchase and delivery, reminder function, health monitoring and management, infectious disease warning, health device compatibility, and health knowledge popularization. The attractive attributes included dialect support, human customer service support, family health sharing, online senior classes, entertainment module, and social interaction support. The indifferent attributes included medical policy inquiry and weather forecast and health advice.

To further clarify the basis of Kano classification and the satisfaction-related performance of each design element, Table 5 present the classification proportions of the 22 health app design elements across five Kano attribute categories, including attractive, one-dimensional, must-be, indifferent, and reverse attributes, together with Q-type results and the corresponding TS, CS, Better, and Worse coefficients. In this study, the Kano classification results were first used to identify the category with the highest proportion for each design element, which then served as the basis for linking the design elements with SEM analysis. Among these categories, must-be, one-dimensional, and attractive attributes were mapped onto three SEM requirement dimensions, namely Basic Requirements, Expected Requirements, and Attractive Requirements. Indifferent and reverse attributes were not included in the SEM requirement dimensions because they had weak or negative explanatory relevance to User Satisfaction. Q-type results indicate inconsistent responses between functional and dysfunctional questions and were therefore not used for SEM mapping.

**Table 5:** Summary of Kano model analysis results.

Function / service	A	O	M	I	R	Q	TS	CS	Classification	Better	Worse
User-friendly design	1.47%	2.65%	65.19%	24.48%	4.42%	1.77%	0.6932	0.4072	Must-be	4.40%	−72.33%
Real-time feedback mechanism	18.58%	46.90%	20.06%	8.55%	2.95%	2.95%	0.8555	0.2684	One-dimensional	69.59%	−71.16%
Advertising reduction	0.29%	2.36%	67.85%	21.24%	5.01%	3.24%	0.7051	0.4661	Must-be	2.89%	−76.53%
Offline use function	0.29%	2.95%	70.80%	20.35%	4.42%	1.18%	0.7405	0.5046	Must-be	3.44%	−78.13%
Voice assistance function	16.52%	46.31%	21.53%	8.55%	1.77%	5.31%	0.8437	0.2478	One-dimensional	67.62%	−73.02%
Dialect support	61.95%	2.06%	1.77%	28.91%	3.83%	1.47%	0.6579	0.3304	Attractive	67.60%	−4.05%
Emergency help-seeking	1.77%	3.83%	59.29%	30.97%	1.47%	2.65%	0.6490	0.2833	Must-be	5.85%	−65.85%
Human customer service support	59.00%	0.88%	1.47%	31.86%	5.60%	1.18%	0.6136	0.2714	Attractive	64.24%	−2.53%
Medical consultation support	24.19%	44.84%	16.22%	7.96%	1.47%	5.31%	0.8526	0.2065	One-dimensional	74.05%	−65.51%
Medicine purchase and delivery	12.98%	50.44%	24.48%	5.01%	2.65%	4.42%	0.8792	0.2597	One-dimensional	68.25%	−80.63%
Medical policy inquiry	20.06%	7.96%	16.81%	44.84%	7.08%	3.24%	0.4483	0.2478	Indifferent	31.25%	−27.63%
Reminder function	19.47%	51.33%	16.22%	7.08%	1.18%	4.72%	0.8702	0.3186	One-dimensional	75.24%	−71.79%
Health monitoring and management	13.86%	46.02%	26.55%	7.08%	1.77%	4.72%	0.8643	0.1947	One-dimensional	64.04%	−77.60%
Infectious disease warning	13.27%	54.87%	21.53%	4.42%	2.36%	3.54%	0.8968	0.3334	One-dimensional	72.41%	−81.19%
Weather forecast and health advice	15.93%	9.14%	14.75%	50.74%	5.90%	3.54%	0.3982	0.3481	Indifferent	27.69%	−26.38%
Health device compatibility	20.06%	45.13%	20.35%	8.26%	2.06%	4.13%	0.8555	0.2478	One-dimensional	69.50%	−69.81%
Family health sharing	68.44%	3.24%	1.18%	20.35%	4.72%	2.06%	0.7287	0.4809	Attractive	76.90%	−4.75%
Privacy protection	0.29%	2.95%	55.75%	36.28%	2.65%	2.06%	0.5900	0.1947	Must-be	3.41%	−61.61%
Online senior classes	56.05%	3.83%	1.47%	30.97%	4.72%	2.95%	0.6136	0.2508	Attractive	64.86%	−5.75%
Entertainment module	61.36%	3.83%	0.59%	25.66%	5.31%	3.24%	0.6579	0.3570	Attractive	71.29%	−4.84%
Social interaction support	64.31%	2.65%	0.29%	25.66%	4.13%	2.95%	0.6726	0.3865	Attractive	72.06%	−3.17%
Health knowledge popularization	20.35%	50.44%	14.45%	8.26%	1.18%	5.31%	0.8525	0.3009	One-dimensional	75.71%	−69.40%

A, attractive attribute; O, one-dimensional attribute; M, must-be attribute; I, indifferent attribute; R, reverse attribute; Q, questionable result. TS, total satisfaction potential; CS, customer satisfaction coefficient. Better and Worse indicate the potential of each design element to increase satisfaction or reduce dissatisfaction.

In this study, Kano categories were not directly treated as SEM latent variables. Rather, the Kano model was used as a preliminary requirement classification method to identify the dominant quality attributes of specific design elements. Based on Kano’s theory of attractive quality, must-be, one-dimensional, and attractive attributes influence User Satisfaction through different mechanisms. Therefore, the design elements classified into these three categories were aggregated into three SEM latent constructs: Basic Requirements, Expected Requirements, and Attractive Requirements. This treatment is supported by recent requirement-driven design studies. For example, Sun et al. proposed the CM Kano model to classify product attributes and support product design improvement by identifying changing customer requirements (33). Tandiono and Rau further integrated the Kano model with QFDE and TRIZ to distinguish the effects of customer requirements and translate them into product improvement priorities (34). Based on these studies, this research further used SEM to examine how the aggregated requirement dimensions influence User Satisfaction, Intention to Use, and User Loyalty. Specifically, Basic Requirements were measured by items classified as must-be attributes, Expected Requirements were measured by items classified as one-dimensional attributes, and Attractive Requirements were measured by items classified as attractive attributes.

**Figure 1:**
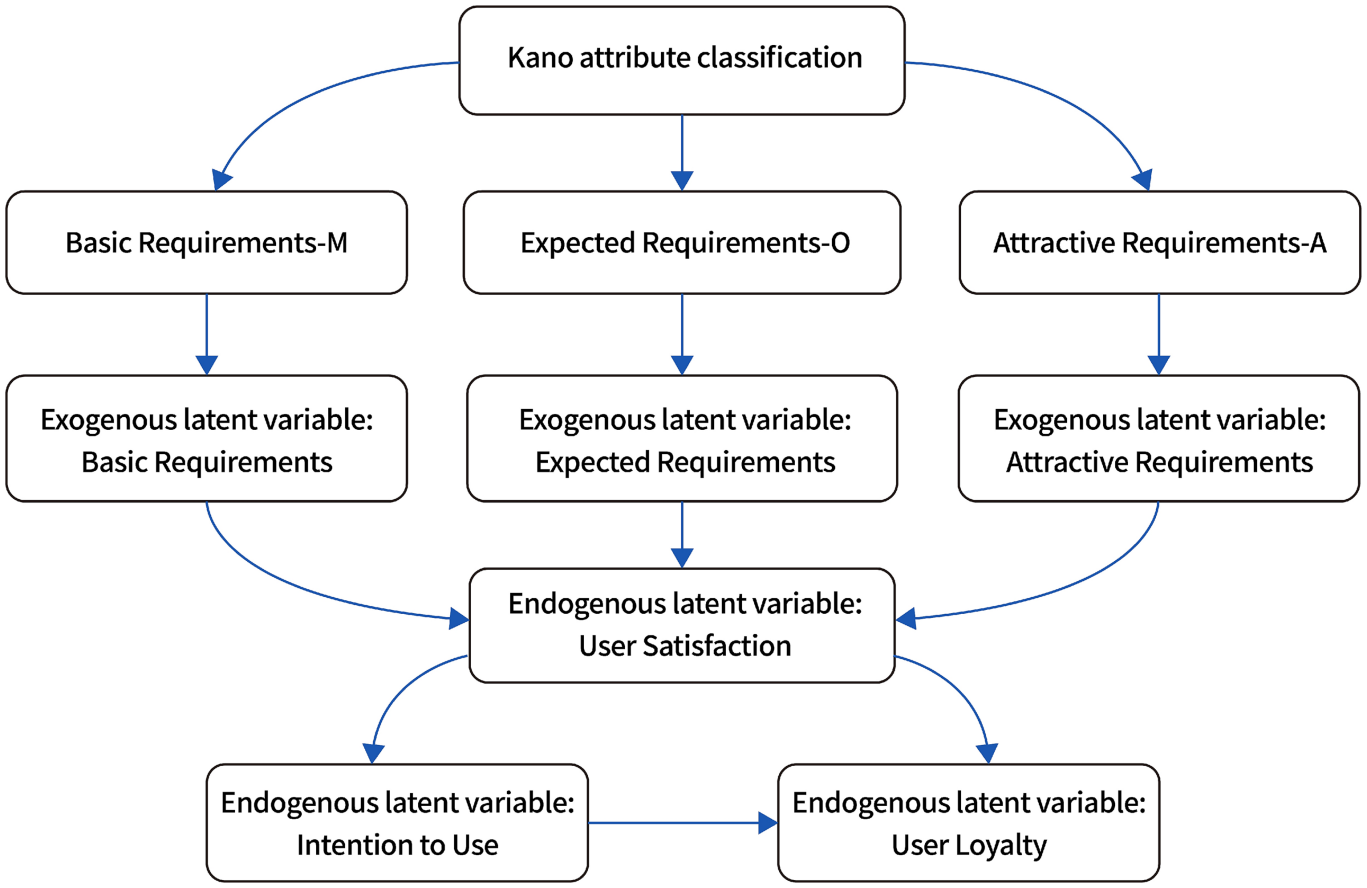
Mapping logic from Kano attribute classification to SEM latent variables.

Based on the Kano classification results shown in Tables 5, 6, Figure 1 illustrates the mapping logic from Kano attribute classification to SEM latent variables.

**Table 6:** Kano classification results of health app design elements.

Kano category	Design elements
M (Must-be / Basic attributes)	1. Interface-friendly design; 3. Advertising reduction; 4. Offline use function; 7. Emergency help-seeking; 18. Privacy protection
O (One-dimensional / Expected attributes)	2. Real-time feedback mechanism; 5. Voice assistance function; 9. Medical consultation support; 10. Medicine purchase and delivery; 12. Reminder function; 13. Health monitoring and management; 14. Infectious disease warning; 16. Health device compatibility; 22. Health knowledge popularization
A (Attractive attributes)	6. Dialect support; 8. Human customer service support; 17. Family health sharing; 19. Online senior classes; 20. Entertainment module; 21. Social interaction support
I (Indifferent attributes)	11. Medical policy inquiry; 15. Weather forecast and health advice

In the SEM analysis, no additional subjective weighting was applied to the Kano categories or design elements. All SEM items were measured using a five-point Likert scale and standardized before model estimation. The relative effects of Basic Requirements, Expected Requirements, and Attractive Requirements were estimated using standardized SEM path coefficients. This conversion process established a quantitative link between Kano-based requirement classification and SEM-based mechanism testing.

### 2.5 Research hypotheses

Based on the mapping logic presented in Figure 1, this study develops a structural model in which Basic Requirements, Expected Requirements, and Attractive Requirements are treated as antecedents of User Satisfaction, while User Satisfaction and Intention to Use explain subsequent behavioral outcomes. The transformation from Kano attributes to SEM constructs is not merely a statistical aggregation. It reflects the theoretical premise that different categories of requirements generate satisfaction through distinct mechanisms. Must-be attributes represent threshold conditions whose absence causes strong dissatisfaction; one-dimensional attributes produce satisfaction in proportion to their performance; and attractive attributes create additional satisfaction by exceeding users’ explicit expectations (27).

For rural older users, these mechanisms should be interpreted within a specific digital-health context. The effectiveness of a health app depends not only on whether functions are available, but also on whether the interface, interaction process, service content, and support mechanisms correspond to users’ sensory, cognitive, technological, and healthcare conditions. Empirical research has shown that older adults frequently encounter serious usability problems arising from complex navigation, overlooked text or icons, limited digital confidence, and age-related cognitive and perceptual barriers (35). Therefore, the three Kano-based requirement constructs are expected to influence User Satisfaction through complementary pathways: Basic Requirements reduce barriers and perceived risk, Expected Requirements strengthen practical utility, and Attractive Requirements enhance emotional inclusion and contextual fit.

*H*1: Basic Requirements have a significant positive effect on User Satisfaction among rural older users.

Basic Requirements include user-friendly interface design, reduced advertising interference, offline use, emergency help-seeking, and privacy protection. These elements constitute the minimum conditions under which rural older users can access and trust a health app. A readable interface and simplified navigation reduce cognitive and visual burden, while offline access responds to unstable network conditions. Emergency assistance and transparent privacy protection further reduce perceived vulnerability and uncertainty. From the perspective of Kano theory, satisfying must-be attributes does not necessarily create strong delight, but failure to satisfy them can rapidly generate frustration, distrust, and rejection. Consequently, when these baseline conditions are adequately provided, users are more likely to evaluate the app as safe, manageable, and dependable, thereby increasing overall satisfaction.

*H*2: Expected Requirements have a significant positive effect on User Satisfaction among rural older users.

Expected Requirements comprise real-time feedback, voice assistance, medical consultation support, medicine purchase and delivery, reminder functions, health monitoring, infectious disease warnings, device compatibility, and health knowledge dissemination. Unlike Basic Requirements, these functions are directly related to the perceived performance and usefulness of the app. Their value becomes greater when they reduce the time, effort, and geographic constraints associated with obtaining healthcare services. For rural older users, voice interaction can compensate for limited typing ability, while consultation, monitoring, reminders, and device connectivity can support continuous health management outside formal medical institutions. Because one-dimensional attributes follow a performance-satisfaction logic, better delivery of these functions should produce a corresponding increase in User Satisfaction.

*H*3: Attractive Requirements have a significant positive effect on User Satisfaction among rural older users.

Attractive Requirements include dialect support, human customer service, family health sharing, online senior classes, entertainment modules, and social interaction. These functions are not indispensable for completing basic health tasks, but they respond to latent needs that are often overlooked in conventional health app design. Dialect and culturally familiar forms of communication can reduce psychological distance from technology; human support can provide reassurance when users encounter difficulties; and family sharing or social functions can strengthen companionship and participation. Research on culturally adapted mHealth interfaces has shown that language, familiar symbols, simple interaction, and context-sensitive design can improve older users’ acceptance and satisfaction (36). Accordingly, Attractive Requirements are expected to generate additional satisfaction by extending the app from a functional health tool to a socially and emotionally supportive service environment.

The preceding hypotheses explain how the three requirement constructs shape users’ immediate evaluation of the health app. However, satisfaction is not the final outcome of the model. In post-adoption research, satisfaction functions as a key psychological mechanism through which users convert their evaluation of system performance into future behavioral intention. Thus, the model further examines whether satisfaction promotes continued use and loyalty.

*H*4: User Satisfaction has a significant positive effect on Intention to Use.

User Satisfaction represents users’ overall assessment of whether the health app meets their expectations regarding ease of use, functional usefulness, health-management support, and emotional acceptability. The expectation-confirmation model of information-system continuance proposes that satisfaction is a principal determinant of continuance intention because positive post-use evaluations strengthen users’ willingness to maintain future use (37). This relationship is particularly important for rural older users, whose continued adoption may be constrained by limited digital experience and anxiety about technology. When an app is perceived as understandable, useful, reliable, and supportive, satisfaction can reduce resistance and reinforce confidence, thereby increasing the intention to use the app in future health-management situations.

*H*5: User Satisfaction has a significant positive effect on User Loyalty.

User Loyalty reflects a more stable and enduring response than a single intention to use. It includes continued preference, repeated use, and willingness to recommend the app to others. Satisfaction provides an essential basis for loyalty formation because repeated positive experiences strengthen trust, preference, and relational attachment. Loyalty theory further indicates that satisfaction is a necessary, although not always sufficient, stage in the development of lasting loyalty (38). In the present context, rural older users who consistently obtain accessible services, practical health support, and respectful interaction are more likely to retain the app as a preferred health-management tool and recommend it within their family or community.

*H*6: Intention to Use has a significant positive effect on User Loyalty.

Intention to Use represents users’ conscious willingness to employ the health app in future situations, whereas User Loyalty captures the consolidation of that willingness into a relatively stable preference and recommendation tendency. A stronger intention indicates that users recognize the app’s value and expect to incorporate it into their health-management routines. Through repeated use, this intention may develop into habitual reliance, lower switching willingness, and positive word of mouth. Accordingly, Intention to Use is expected to serve as a proximal behavioral mechanism linking satisfaction with longer-term loyalty. This path also clarifies that loyalty does not emerge directly from design attributes alone, but develops through a sequential process in which requirement fulfillment improves satisfaction, satisfaction strengthens intention, and intention contributes to continued preference.

Taken together, H1-H3 explain the differentiated effects of Kano-based requirement categories on User Satisfaction, while H4-H6 describe the subsequent behavioral pathway from satisfaction to use intention and loyalty. This sequence establishes a complete theoretical chain from design requirement classification to post-adoption behavior and provides the conceptual basis for SEM testing.

## 3 Results

Before the data analysis, the valid data in the questionnaire were first analyzed for reliability. According to the results in Table 7, the reliability coefficients of each latent variable were high, indicating that the questionnaire data of this study had good reliability and could provide a solid foundation for subsequent analysis.

**Table 7:** Reliability indicators for each dimension.

Latent constructs	Number of questions	Cronbach’s α
Basic Requirements	5	0.875
Expected Requirements	9	0.9
Charm demand	6	0.877
User Satisfaction	5	0.899
Intent to use	4	0.917
User Loyalty	3	0.823
Full Scale	32	0.929

According to Table 7, Cronbach’s *α* values for all dimensions exceeded 0.8, meeting the reliability criteria, indicating high reliability of the survey data and providing effective support for subsequent structural equation model (SEM) analysis.

To further test the structural validity of the questionnaire, confirmatory factor analysis (CFA) was conducted in this study. Table 8 shows factor loadings, combined reliability (CR), and mean extraction variance (AVE) values for all latent variables. The results show that factor loadings for all latent variables are greater than 0.5, CR values are greater than 0.6, and AVE values are greater than 0.5, meeting the criteria for confirmatory factor analysis. The questionnaire has good structural validity.

**Table 8:** Confirmatory factor analysis results.

Latent variable	Observation indicator code	Observation indicator name	Factor loading	CR	AVE
Basic requirements	BR1	User-friendly interface design	0.775	0.877	0.592
	BR2	Advertising reduction	0.838		
	BR3	Offline use function	0.869		
	BR4	Emergency help-seeking	0.718		
	BR5	Privacy protection	0.622		
Expected requirements	ER1	Real-time feedback mechanism	0.776	0.904	0.516
	ER2	Voice assistance function	0.709		
	ER3	Medical consultation support	0.784		
	ER4	Medicine purchase and delivery	0.720		
	ER5	Reminder function	0.689		
	ER6	Health monitoring and management	0.867		
	ER7	Infectious disease warning	0.545		
	ER8	Health device compatibility	0.587		
	ER9	Health knowledge popularization	0.732		
Attractive requirements	AR1	Dialect support	0.814	0.879	0.548
	AR2	Human customer service support	0.736		
	AR3	Family health sharing	0.772		
	AR4	Online senior classes	0.678		
	AR5	Entertainment module	0.753		
	AR6	Social interaction support	0.678		
User satisfaction	US1	Overall satisfaction with the health app	0.795	0.907	0.661
	US2	Satisfaction with functional usefulness	0.803		
	US3	Satisfaction with ease of use	0.771		
	US4	Satisfaction with health management support	0.828		
	US5	Satisfaction compared with expectations	0.866		
User loyalty	UL1	Willingness to continue using the health app	0.852	0.829	0.620
	UL2	Willingness to recommend the health app to others	0.793		
	UL3	Preference for continuing to use this health app	0.710		
Intention to use	IU1	Intention to use the health app in the future	0.819	0.920	0.743
	IU2	Willingness to use the app for daily health management	0.870		
	IU3	Intention to use the app when health requirements arise	0.894		
	IU4	Willingness to increase use of the health app	0.863		

As shown from the data in Table 8, factor loadings for all latent variables are significantly higher than 0.5, and the combined reliability and average extracted variance meet the criteria, indicating that the structural validity has been fully validated.

Next, a correlation analysis was conducted in this study to further explore the relationships among the latent variables. The analysis results in Table 9 indicated that there was a significant positive correlation (*p* < 0.01) among all latent variables. Specifically, there is a significant positive correlation between the Expected Requirements and theAttractive Requirements for User Satisfaction, and there is also a significant positive correlation between the Attractive Requirements and the intention to use and User Loyalty.

**Table 9:** Results of correlation analysis.

Variables	Basic requirements	Expected requirements	Attractive requirements	User satisfaction	Intent to use	User loyalty
Basic Requirements	1					
Expected Requirements	0.228**	1				
Attractive Requirements	0.327**	0.560**	1			
User Satisfaction	0.297**	0.421**	0.498**	1		
Intent to Use	0.259**	0.418**	0.431**	0.303**	1	
User Loyalty	0.242**	0.276**	0.334**	0.305**	0.236**	1

**p < 0.01.

As can be seen from the above table, the correlation analysis shows that there is a significant positive correlation among all variables (*p* < 0.01). Among them, the Expected Requirements (*r* = 0.560, *p* < 0.01) and the Attractive Requirements (*r* = 0.498, *p* < 0.01) were both positively correlated with User Satisfaction, and the Basic Requirements (*r* = 0.297, *p* < 0.01) was also positively correlated with User Satisfaction. Attractive Requirements were significantly positively correlated with use intention (*r* = 0.431, *p* < 0.01) and User Loyalty (*r* = 0.334, *p* < 0.01).

The results of the reliability test, confirmatory factor analysis, and correlation analysis indicated that the questionnaire data of this study were reliable, structurally valid, and there was a significant correlation among the latent variables. Based on this, the study will further explore how design elements can optimize the design of health apps by influencing the satisfaction, intention to use, and loyalty of older users through meeting their Basic Requirements, Expected Requirements, and appealing needs.

**Figure 2:**
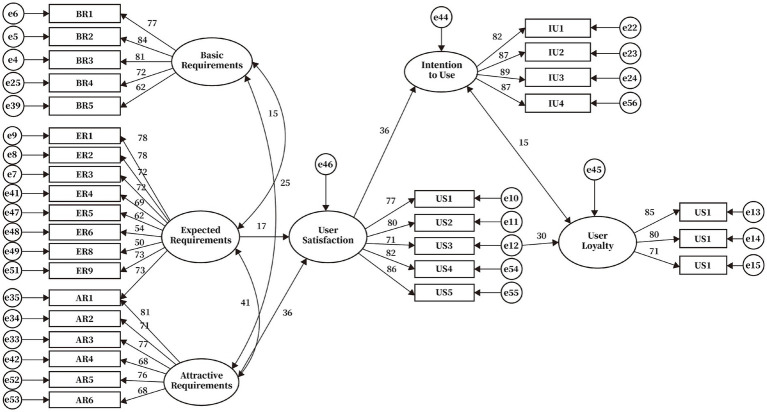
Initial structural equation model.

**Figure 3:**
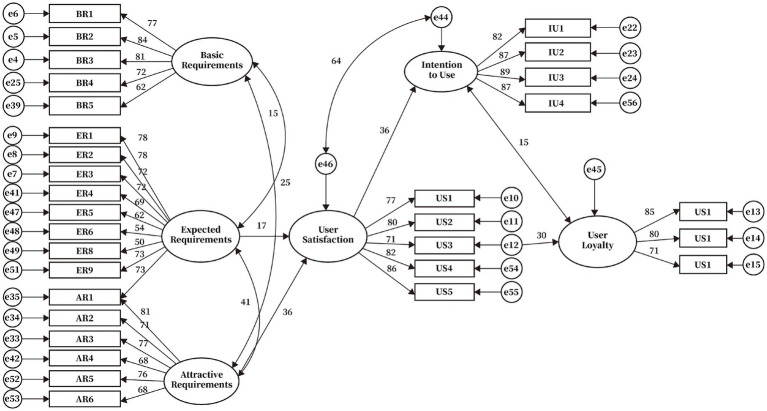
Modified model.

In this study, a structural equation model (SEM) was used for analysis in order to further test the causal relationship between the design elements of health apps and user behavior. First, an initial model (Figure 2) was constructed and optimized (Figure 3) to improve the model’s fit. By analyzing the modification index (MI) of the model, it was found that the “Expected Requirements” item (e49, e50), the residual of User Satisfaction (e46), and the residual of Intention to Use (e44) had higher MI values. Based on this finding, the model was modified by connecting the relevant paths of these variables, further enhancing the model’s fit.

Fit metrics. Model fit assessment is a key metric for testing a model’s interpretive ability. The following results were obtained based on common fitting metrics such as chi-square ratio of degrees of freedom, RMSEA, GFI, etc. (as shown in Table 10).

**Table 10:** Fitting metrics.

Common indicators	Chi-square degrees of freedom ratio χ^2^/df	RMSEA	GFI	NFI	IFI	TLI	CFI
Criteria for judgment	<5	<0.10	>0.8	>0.8	>0.8	>0.8	>0.8
value	1.341	0.033	0.897	0.904	0.974	0.971	0.974

χ^2^/df, chi-square divided by degrees of freedom; RMSEA, root mean square error of approximation; GFI, goodness-of-fit index; NFI, normed fit index; IFI, incremental fit index; TLI, Tucker-Lewis index; CFI, comparative fit index.

In summary, all six research hypotheses were supported. Table 11 provides a consolidated overview of the hypothesis testing results, including the standardized path coefficients, significance levels, and support outcomes.

**Table 11:** Summary of hypothesis testing results.

Hypothesis	Path	Standardized coefficient β	*p*-value	Result
H1	Basic Requirements → User Satisfaction	0.297	<0.01	Supported
H2	Expected Requirements → User Satisfaction	0.421	<0.01	Supported
H3	Attractive Requirements → User Satisfaction	0.498	<0.01	Supported
H4	User Satisfaction → Intention to Use	0.303	<0.01	Supported
H5	User Satisfaction → User Loyalty	0.305	<0.01	Supported
H6	Intention to Use → User Loyalty	0.236	<0.01	Supported

β refers to the standardized path coefficient. *p* < 0.01 indicates that the path is statistically significant.

The results indicate that all six hypotheses were supported. Among the three requirement-related constructs, Attractive Requirements showed the strongest effect on User Satisfaction, followed by Expected Requirements and Basic Requirements. This suggests that emotionally supportive and context-adaptive functions play an important role in improving rural older users’ satisfaction with health apps.

## 4 Discussion

This study examined how different categories of design requirements influence rural older users’ satisfaction, intention to use, and loyalty toward health apps. The results show that Basic Requirements, Expected Requirements, and Attractive Requirements all had significant positive effects on User Satisfaction. User Satisfaction further had significant positive effects on both Intention to Use and User Loyalty, while Intention to Use also positively influenced User Loyalty.

More importantly, the integrated Kano–SEM results indicate that these three requirement categories contribute to satisfaction through different mechanisms and with different levels of influence. The standardized path coefficients show that Attractive Requirements had the strongest effect on User Satisfaction (*β* = 0.498), followed by Expected Requirements (*β* = 0.421) and Basic Requirements (*β* = 0.297). This pattern means that Kano classification should not be interpreted merely as a static list of design priorities. When combined with SEM, the model explains how classified design requirements are converted into satisfaction and subsequently into intention to use and loyalty. The findings therefore reveal a layered behavioral mechanism in which usability and trust establish the conditions for adoption, functional usefulness strengthens perceived value, and emotional and contextual adaptation produces additional satisfaction and supports longer-term engagement.

### 4.1 Interpretation of the main findings

The first important finding is that Basic Requirements significantly influenced User Satisfaction. For rural older users, interface friendliness, clear icons, readable fonts, simple operation, privacy protection, reduced advertising interference, offline use, and emergency help-seeking are not auxiliary features but threshold conditions for app acceptance. Older adults often experience declines in vision, memory, attention, and information-processing speed. Consequently, complex navigation, small text, unclear feedback, and excessive operational steps may increase cognitive load and quickly generate frustration or distrust. From the perspective of the Kano model, these requirements primarily prevent dissatisfaction; from the SEM perspective, however, their significant positive path to satisfaction shows that removing basic barriers also creates a necessary foundation on which higher-level functions can operate effectively.

The second finding is that Expected Requirements had a significant positive effect on User Satisfaction. Functions such as voice assistance, online consultation, medication reminders, health monitoring, device compatibility, and health knowledge dissemination directly support daily health-management tasks. Their effect can be explained by the limited accessibility of medical resources, transportation barriers, and uneven digital support in rural areas. These functions increase the practical usefulness of health apps by reducing the time, effort, and uncertainty involved in obtaining health information and services. The SEM coefficient further confirms the one-dimensional logic of the Kano model: as the performance and accessibility of these functions improve, users’ evaluation of the app also improves. Thus, Expected Requirements act as the principal functional mechanism through which health apps generate perceived value in rural healthcare contexts.

The third finding is that Attractive Requirements produced the strongest effect on User Satisfaction. Dialect support, human customer service, family health sharing, online senior classes, entertainment modules, and social interaction are not always considered essential before use, but they can generate a substantial increase in satisfaction once provided. This result may be attributed to the social and cultural conditions of rural aging. Some rural older users have limited proficiency in standard Mandarin, low digital confidence, weak access to immediate technical assistance, and a strong reliance on family and familiar interpersonal networks. Attractive Requirements therefore reduce psychological distance from technology and create a sense of being understood, supported, and socially connected. Their strong SEM effect suggests that, in this context, emotional inclusion and local adaptation are not peripheral benefits but important drivers of positive user experience.

The fourth finding is that User Satisfaction significantly promoted both Intention to Use and User Loyalty, while Intention to Use also had a positive effect on loyalty. These paths extend the interpretation beyond Kano-based requirement classification by showing how design outcomes develop into behavioral consequences. Satisfaction functions as the central conversion mechanism: when users perceive an app as usable, useful, trustworthy, and emotionally acceptable, they are more willing to continue using it and to recommend it to others. The positive relationship between Intention to Use and User Loyalty further indicates that repeated willingness may gradually become stable preference and recommendation behavior. Therefore, the integrated model identifies a sequential pathway from requirement fulfillment to satisfaction, from satisfaction to use intention, and from use intention to loyalty.

### 4.2 Comparison with previous studies

The findings are consistent with prior studies on age-friendly interface design, which identify readable fonts, clear icons, appropriate color contrast, simplified navigation, and reduced cognitive load as key factors in older adults’ adoption of mobile health technologies (35, 39, 40). The significant path from Basic Requirements to User Satisfaction supports this body of research. However, the present study adds a more differentiated explanation: basic usability features are not only interface-level recommendations but a latent requirement construct with a statistically verified effect on satisfaction. This distinction is important because it links concrete design elements to an overall behavioral mechanism rather than treating them as isolated usability guidelines.

The results also correspond with technology acceptance research, which emphasizes perceived ease of use and perceived usefulness as major predictors of digital technology adoption (41, 42). In the present study, Expected Requirements translate perceived usefulness into specific rural health-management services, including consultation, reminders, monitoring, and voice interaction. The findings therefore support existing acceptance models while also extending them by showing that usefulness in rural older adult health apps is context dependent. It must be embodied in functions that compensate for limited healthcare accessibility, low digital literacy, and weak service connectivity rather than remaining an abstract perception of technological performance.

The strongest effect of Attractive Requirements is broadly consistent with research on emotional design, personalized services, and social engagement in digital health, which shows that such attributes can enhance satisfaction and continued use (43, 44). Nevertheless, this study advances the discussion by identifying the specific form of emotional value in rural settings. Here, emotional value is closely tied to dialect communication, family participation, familiar support channels, and culturally accessible interaction. In contrast to studies that treat attractive attributes mainly as innovative or personalized features, the present findings suggest that their value lies in reducing technology-related anxiety and compensating for deficits in social and service support. This contextual interpretation helps explain why Attractive Requirements exerted a stronger effect than the other two categories.

### 4.3 Differences from previous studies and research innovations

Although the findings are partly consistent with previous research, this study differs in several respects. First, it focuses specifically on rural older users rather than treating older adults as a homogeneous group. Rural users’ app experiences are shaped by the combined effects of digital literacy, healthcare accessibility, network conditions, dialect communication, and family support. Prior evidence has shown that older adults’ digital health literacy is unevenly distributed and influenced by residence, internet-use experience, training opportunities, and broader participation conditions (45). By incorporating these contextual factors, the study provides a more precise explanation of technology adoption in rural aging communities.

Second, the study moves beyond the fragmented treatment of individual design elements. Previous research often examines font size, navigation, voice interaction, privacy, or reminders separately. The Kano model used here first organizes these elements into Basic, Expected, and Attractive Requirements, while SEM then estimates their relative effects on satisfaction and subsequent behavioral outcomes. The combination therefore produces both a design taxonomy and an explanatory model, allowing the study to identify not only which features matter but also how strongly different requirement levels shape user responses.

Third, the methodological integration of Kano and SEM extends prior Kano-based studies that usually stop at classification and prioritization (46). The present study converts Kano-derived requirement categories into latent constructs and empirically tests their relationships with User Satisfaction, Intention to Use, and User Loyalty. This integration connects micro-level feature identification with macro-level behavioral explanation. It also reveals that the practical priority of a requirement cannot be inferred only from its Kano category; its behavioral importance must also be assessed through the magnitude and significance of SEM paths.

Fourth, the finding that Attractive Requirements exerted the strongest effect challenges an exclusively usability-oriented view of age-friendly health app design. Interface simplification remains necessary, but it is not sufficient to sustain engagement. For rural older users, local language, human assistance, family connection, and social participation can generate a stronger experiential response than basic operational improvements alone. This finding introduces contextual and relational value into the theoretical explanation of age-friendly digital health services.

### 4.4 Academic contributions of this study

This study contributes theoretically by establishing an integrated Kano–SEM framework that links design requirement classification to behavioral outcomes. The framework differentiates three roles within the same explanatory system: Basic Requirements establish the usability and trust threshold, Expected Requirements deliver functional and health-management value, and Attractive Requirements generate emotional and contextual value. SEM then clarifies how these roles influence satisfaction and how satisfaction develops into intention and loyalty. This advances previous research that relied either on general technology acceptance variables or on Kano-based feature classification alone. The main theoretical contribution is therefore not simply the use of two methods, but the construction of a cross-level mechanism connecting design attributes, perceived experience, and continued behavioral response.

The findings also refine the theoretical understanding of satisfaction in rural digital health. Satisfaction is shown to be more than a direct evaluation of interface quality or functional performance; it integrates usability, usefulness, trust, emotional inclusion, and contextual fit. The stronger path of Attractive Requirements demonstrates that satisfaction among rural older users is partly relational and culturally situated. This extends conventional acceptance models by showing that local language, family participation, and accessible human support can become central components of technology evaluation when users face both digital and healthcare disadvantages.

From a practical perspective, the study provides a layered design strategy for rural older adult health apps. Basic Requirements should be treated as the usability and trust baseline, Expected Requirements as the core health-management service layer, and Attractive Requirements as the emotional and contextual enhancement layer. This structure enables developers, healthcare institutions, and community service providers to translate empirical findings into coordinated priorities rather than isolated feature improvements. It also suggests that rural digital health services should be evaluated not only by technical accessibility but by their capacity to connect users with healthcare resources, family support, and culturally familiar forms of interaction.

### 4.5 Limitations and future research directions

This study provides useful insights, but several limitations should be acknowledged. First, the sample was limited to older users in rural areas of Guangdong Province. Guangdong is a meaningful research setting because it combines rapid digital development with rural aging and uneven access to health resources. However, regional differences in economic development, medical infrastructure, dialects, cultural habits, and digital literacy may influence how rural older users perceive and use health apps. Therefore, future studies should include samples from multiple provinces and different rural contexts to further test the generalizability and robustness of the findings.

Second, this study mainly relied on questionnaire data. Although the questionnaire was designed based on user requirement sets and statistical tests supported its reliability and validity, self-reported data may still be affected by memory bias, social desirability bias, or inaccurate estimation of app usage behavior. Future research could combine questionnaire data with app usage logs, behavioral tracking, field observation, or longitudinal follow-up to obtain more objective evidence on actual use behavior and health management outcomes.

Third, although this study verified the relationships among design requirements, satisfaction, Intention to Use, and loyalty, the interaction mechanisms among different design elements still require deeper analysis. For example, basic usability may strengthen the effect of expected functions, while attractive features may become more influential after basic operational barriers are reduced. Future studies could further explore these interaction effects and examine whether age, education level, digital literacy, chronic disease status, or family support moderates the relationship between design optimization and user behavior.

Finally, future research should further connect design optimization with measurable health outcomes. This study mainly examined satisfaction, Intention to Use, and loyalty, which are important behavioral indicators. However, whether optimized health apps can improve medication adherence, health monitoring frequency, health knowledge acquisition, or chronic disease self-management still needs stronger empirical verification. Future studies could conduct intervention-based or longitudinal research to evaluate whether age-friendly health app design can produce sustained improvements in rural older users’ health management behavior and health outcomes.

## 5 Conclusion

This study verified the significant impact of health app design elements on the behavior of older users in rural areas through structural equation modeling analysis. The findings show that the satisfaction of Basic, Expected, and Attractive Requirements not only significantly increases the satisfaction of older users, but also further influences their use intention and User Loyalty. This finding provides theoretical support for the design of health apps and valuable guidance for future age-friendly design practices. More importantly, the results suggest that health app optimization should not be understood only as a product-level design issue, but also as a broader process involving user differentiation, regional adaptation, stakeholder collaboration, and integration with local healthcare services.

From the perspective of design studies, this research not only verified the multi-level structure of the requirements of middle-aged and older users in the design of health apps, but also provided practical suggestions on how to transform these requirements into specific design elements. First of all, the satisfaction of Basic Requirements should be at the core of the design, and the demand for interface friendliness and privacy protection among older users is particularly urgent. APP design should focus on a simple and user-friendly interface, as well as privacy protection measures to ensure the security of user data. These basic functions are directly related to the user experience and trust of older users. Secondly, the satisfaction of Expected Requirements can further enhance user experience and satisfaction, especially in the context of intelligent health management. Functional needs such as voice assistance, medical consultation, and health monitoring can effectively fill the gaps in traditional health management for older adults. Therefore, the design of health apps should focus on providing more practical and efficient support for older users in terms of technical implementation and functional expansion. In addition, the satisfaction of the demand for charm provides direction for design innovation. In today’s highly competitive health APP market, additional features such as dialect support, social interaction, and entertainment are not basic requirements, but they can enhance the APP’s appeal by boosting the user’s emotional experience. It can be seen that the design of health apps should incorporate these “surprise” features innovatively based on the cultural background and emotional needs of older users in rural areas, thereby enhancing users’ long-term loyalty.

Although this study focuses on rural older users, some insights about health app optimization may also be extended to general users to a certain extent. For example, simplified interfaces, clear information architecture, privacy protection, voice assistance, and proactive reminders are not only useful for older users, but can also improve the experience of users with low digital literacy, users with physical or cognitive limitations, and users in medically underserved areas. However, these findings should not be directly generalized to all users without adjustment. Different user groups may place different emphasis on Basic, Expected, and Attractive Requirements. For general adult users, functions related to health monitoring efficiency, data integration, and personalized management may be more important, whereas dialect support, localized content, and family health sharing are particularly relevant to rural older users. Therefore, the design framework proposed in this study has transferable value, but its application should be calibrated according to specific user groups and usage contexts.

The findings also indicate that health app optimization priorities may differ across Chinese regions. In regions with relatively mature digital infrastructure and higher digital literacy, optimization can place greater emphasis on expected and attractive needs, such as remote medical consultation, health device integration, personalized health management, and data security. In less developed rural areas or regions with weak network infrastructure, Basic Requirements such as offline use, simplified navigation, voice guidance, low-bandwidth operation, and readable health information should be given priority. In areas with strong dialect use or distinctive local cultural habits, language adaptation and localized health content should also be considered important design priorities. Therefore, health app optimization in China should not adopt a single uniform model, but should be adjusted according to regional differences in digital infrastructure, healthcare accessibility, older users’ digital literacy, and local health service conditions.

In terms of responsibility, the optimization of health apps should not rely solely on app developers or technology companies. It requires the joint participation of multiple stakeholders. App developers and interaction designers should be responsible for transforming older users’ needs into accessible, usable, and emotionally inclusive product functions. Healthcare institutions, village clinics, township hospitals, and community health workers should provide professional medical content, health service connection, and practical support for rural users. Public health authorities and government departments should establish standards for accessibility, data security, privacy protection, and digital inclusion. Older users and their family members should also be involved in usability testing and feedback collection, so that the final design can better correspond to real-life health management scenarios.

To better support local healthcare services, health app development should be connected with existing local medical and public health systems rather than being developed as an isolated digital product. In rural areas, health apps should be linked with village doctor networks, township hospitals, family doctor services, chronic disease management programs, and local public health activities. Functions such as medication reminders, health monitoring, infectious disease warnings, online consultation, medical policy inquiry, and family health sharing should be adapted to local healthcare workflows. Health information should also be presented in plain language and supported by voice interaction or dialect-based communication when necessary. Through such localization, health apps can become part of the local healthcare support network and better serve the daily health management requirements of rural older users.

Overall, this study provides important theoretical basis and practical guidance for the age-friendly design of health apps from the perspective of design studies. By meeting the Basic, Expected, and Attractive Requirements of older users, the user experience and long-term loyalty can be significantly enhanced. Future research should continue to deepen the integration of age-friendly design and technology application, further enhance the health management effect of the older adults in rural areas, and promote the wide application of health apps in rural areas. Future studies should also examine how age-friendly design principles can be adapted to different regions, user groups, and healthcare service systems, and further explore collaborative mechanisms among designers, developers, healthcare providers, public authorities, local communities, older users, and their families.

## Data Availability

The original contributions presented in the study are included in the article/Supplementary material, further inquiries can be directed to the corresponding author.
