# Supplementary material for: Design optimization of health apps for rural older adults: enhancing usability and health effects based on Kano model and SEM method
Source: Front Public Health. 2026 Jul 3;14:1810287. doi: 10.3389/fpubh.2026.1810287 (PMC13375450; doi:10.3389/fpubh.2026.1810287)
Supplement: Supplementary file 1 [file Supplementary_file_1.docx]

**Appendix**

**Appendix A. Kano Functional-and-Dysfunctional Question Items**

For each design element, respondents answered both the functional and dysfunctional questions using the following five-response Kano scale.

**Table A1. Kano Functional-and-Dysfunctional Question Items**

| **No.** | **Category** | **Design element** | **Positive Kano item** | **Negative Kano item** | **Main literature basis** |
| --- | --- | --- | --- | --- | --- |
| 1 | Interface and interaction design | Interface-friendly design | How would you feel if the health app provides large fonts, clear icons, simple layout, and high color recognition? | How would you feel if the health app does not provide large fonts, clear icons, simple layout, or high color recognition? | Salman et al., 2018; Khamaj & Ali, 2024; Wang et al., 2022 |
| 2 | Interface and interaction design | Real-time feedback mechanism | How would you feel if the app provides clear voice or visual feedback after each operation? | How would you feel if the app does not provide clear feedback after each operation? | Kalimullah & Sushmitha, 2017; Morey et al., 2019 |
| 3 | Interface and interaction design | Reduced advertising | How would you feel if the app reduces irrelevant or misleading advertisements? | How would you feel if the app contains frequent irrelevant or misleading advertisements? | Nouri et al., 2018; Wang et al., 2022 |
| 4 | Interface and interaction design | Offline use features | How would you feel if the app supports basic health knowledge, disease-prevention guidance, and reminders under unstable network conditions? | How would you feel if the app cannot provide basic information or reminders when the network is unstable or unavailable? | Peng et al., 2016; Ming et al., 2025 |
| 5 | Voice and language support | Voice assistance features | How would you feel if the app provides voice reading, voice search, voice input, and voice reminders? | How would you feel if the app does not provide voice reading, voice search, voice input, or voice reminders? | Khamaj & Ali, 2024; Li et al., 2021 |
| 6 | Voice and language support | Dialect support | How would you feel if the app supports dialect recognition and dialect-based voice interaction? | How would you feel if the app only supports standard Mandarin and does not support dialects? | Fryer et al., 2012; Peng et al., 2016 |
| 7 | Emergency and service support | Urgent help | How would you feel if the app provides one-click emergency help and automatically sends your location to family members, village doctors, or nearby medical institutions? | How would you feel if the app does not provide one-click emergency help or location sharing? | Chen et al., 2023; Valkonen et al., 2025 |
| 8 | Emergency and service support | Human customer service support | How would you feel if the app provides human customer service through phone or online chat to solve usage or health inquiry problems? | How would you feel if the app does not provide human customer service support? | Ahmad et al., 2022; Lee et al., 2020 |
| 9 | Medical service support | Medical consultation support | How would you feel if the app connects local village doctors with remote specialists for video consultation and online medical advice? | How would you feel if the app does not provide online medical consultation support? | Peng et al., 2016; Lin et al., 2023 |
| 10 | Medical service support | Medicine purchase and delivery | How would you feel if the app supports online medicine purchase and home delivery from local pharmacies? | How would you feel if the app does not support online medicine purchase or delivery? | Chen et al., 2023; Lin et al., 2023 |
| 11 | Medical service support | Medical policy inquiry | How would you feel if the app helps you understand medical insurance, reimbursement, and related health policies? | How would you feel if the app does not provide medical policy inquiry services? | Wu & Liu, 2020; Ming et al., 2025 |
| 12 | Health management support | Reminder feature | How would you feel if the app provides medication, medical appointment, physical examination, and vaccination reminders through voice, vibration, or pop-ups? | How would you feel if the app does not provide medication, appointment, examination, or vaccination reminders? | Li et al., 2021; Valkonen et al., 2025 |
| 13 | Health management support | Health monitoring and management | How would you feel if the app provides step counting, dietary guidance, sleep analysis, and health management functions? | How would you feel if the app does not provide health monitoring or management functions? | Yu & Huang, 2020; Lin et al., 2023 |
| 14 | Health management support | Infectious disease warnings | How would you feel if the app provides timely infectious disease warnings and protection guidelines? | How would you feel if the app does not provide infectious disease warnings or protection guidelines? | Chen et al., 2023; Ge et al., 2025 |
| 15 | Health management support | Weather forecasts and health advice | How would you feel if the app provides weather forecasts and health advice based on weather conditions? | How would you feel if the app does not provide weather forecasts or weather-related health advice? | Ming et al., 2025; Valkonen et al., 2025 |
| 16 | Device and data support | Health device compatibility | How would you feel if the app can connect with common health monitoring devices and synchronize health data in real time? | How would you feel if the app cannot connect with common health monitoring devices? | Nouri et al., 2018; Chen et al., 2023 |
| 17 | Device and data support | Family health sharing | How would you feel if the app allows family members to remotely view your health data with permission? | How would you feel if the app does not support family health sharing? | Lee et al., 2020; Chen et al., 2023 |
| 18 | Device and data support | Privacy protection | How would you feel if the app provides simple and understandable privacy settings to protect your health data and personal information? | How would you feel if the app does not provide clear privacy protection or data security settings? | Nouri et al., 2018; Chen et al., 2023 |
| 19 | Learning and social support | **Online older adult classes** | How would you feel if the app provides video tutorials and voice explanations to teach smartphone and app use? | How would you feel if the app does not provide learning support for **older adult users**? | Burma, 2023; Ahmad et al., 2022 |
| 20 | Learning and social support | Entertainment module | How would you feel if the app provides opera, music, crosstalk, dance videos, and other entertainment content? | How would you feel if the app does not provide entertainment content for daily life? | Yu & Huang, 2020; Valkonen et al., 2025 |
| 21 | Learning and social support | Social interaction support | How would you feel if the app provides a communication platform for older adults to reduce loneliness and enhance social interaction? | How would you feel if the app does not provide social interaction functions? | Chen et al., 2023; Lee et al., 2020 |
| 22 | Learning and social support | Health knowledge sharing | How would you feel if the app provides health knowledge through videos, text, and voice explanations? | How would you feel if the app does not provide health knowledge sharing functions? | Li et al., 2021; Wang et al., 2022 |

**Appendix B. Questionnaire on Health Apps for Rural Older Adult Users**

**Dear Participant:**

Thank you for participating in this survey. This questionnaire aims to collect your actual experiences and usage of digital health technologies. All information you provide will be kept strictly confidential and used only for academic research. You do not need to worry about personal privacy, as all responses will be processed anonymously and the data will be used solely for research analysis. When completing the questionnaire, please answer according to your actual experiences and feelings. There are no right or wrong answers. Your truthful responses are very important for helping us understand the needs and barriers of **rural older adult users** when using health apps. We sincerely appreciate your time, understanding, and support.

**Instructions:** For single-choice questions, please select one answer. For multiple-choice questions, you may select more than one answer. For Kano items, please select the option in each row that best matches your feelings.

**I. Basic Information**

**1. Your gender:**

○ Male ○ Female

**2. Your age:**

○ 60-65 years old ○ 65-70 years old ○ 70-75 years old ○ 75-80 years old ○ Over 80 years old

**3. Your current place of residence:**

○ Rural area ○ Township

**4. How long have you used a smartphone or smart device?**

○ Less than 1 year ○ 1-3 years ○ 3 years or more

**5. Your chronic disease status:**

○ None ○ Hypertension ○ Diabetes ○ Other

**6. Which health apps have you usually accessed or used?**

☐ DXY Doctor ☐ 114 Health ☐ Chunyu Doctor ☐ Baidu Doctor ☐ Ping An Health ☐ Good Doctor Online ☐ Ping An Good Doctor ☐ Ali Health ☐ Other

**II. Kano Questionnaire on Functional Requirements for Health Apps**

Please evaluate the following functions under two conditions: when the function is provided and when the function is not provided, based on your true feelings.

**7. Interface-friendly design**

| **Item** | **I dislike it** | **I can tolerate it** | **Neutral** | **It should be that way** | **I like it** |
| --- | --- | --- | --- | --- | --- |
| If a health app provides large fonts, large icons, a clear layout, high color recognition, and reduced operational complexity, how would you feel? | ○ | ○ | ○ | ○ | ○ |
| If a health app does not provide large fonts, large icons, a clear layout, or an easy-to-recognize interface, how would you feel? | ○ | ○ | ○ | ○ | ○ |

**8. Real-time feedback mechanism**

| **Item** | **I dislike it** | **I can tolerate it** | **Neutral** | **It should be that way** | **I like it** |
| --- | --- | --- | --- | --- | --- |
| If a health app provides clear text or voice prompts when an operation succeeds or fails, helping you reduce operational confusion, how would you feel? | ○ | ○ | ○ | ○ | ○ |
| If a health app does not provide clear feedback prompts when an operation succeeds or fails, how would you feel? | ○ | ○ | ○ | ○ | ○ |

**9. Advertising reduction**

| **Item** | **I dislike it** | **I can tolerate it** | **Neutral** | **It should be that way** | **I like it** |
| --- | --- | --- | --- | --- | --- |
| If a health app reduces irrelevant advertisements, pop-up advertisements, or misleading advertisements, how would you feel? | ○ | ○ | ○ | ○ | ○ |
| If a health app contains many irrelevant advertisements, pop-up advertisements, or misleading advertisements, how would you feel? | ○ | ○ | ○ | ○ | ○ |

**10. Offline use function**

| **Item** | **I dislike it** | **I can tolerate it** | **Neutral** | **It should be that way** | **I like it** |
| --- | --- | --- | --- | --- | --- |
| If a health app allows you to view basic health knowledge, disease prevention guidelines, and reminder information when the network is unstable or unavailable, how would you feel? | ○ | ○ | ○ | ○ | ○ |
| If a health app cannot use any related basic functions when the network is unstable or unavailable, how would you feel? | ○ | ○ | ○ | ○ | ○ |

**11. Voice assistance function**

| **Item** | **I dislike it** | **I can tolerate it** | **Neutral** | **It should be that way** | **I like it** |
| --- | --- | --- | --- | --- | --- |
| If a health app provides voice reading, voice search, voice input, and voice reminders, how would you feel? | ○ | ○ | ○ | ○ | ○ |
| If a health app does not provide voice reading, voice search, voice input, or voice reminders, how would you feel? | ○ | ○ | ○ | ○ | ○ |

**12. Dialect support**

| **Item** | **I dislike it** | **I can tolerate it** | **Neutral** | **It should be that way** | **I like it** |
| --- | --- | --- | --- | --- | --- |
| If a health app supports multiple dialect recognition or dialect voice services to reduce operational barriers caused by non-standard Mandarin, how would you feel? | ○ | ○ | ○ | ○ | ○ |
| If a health app does not support dialect recognition or dialect voice services, how would you feel? | ○ | ○ | ○ | ○ | ○ |

**13. Emergency help-seeking**

| **Item** | **I dislike it** | **I can tolerate it** | **Neutral** | **It should be that way** | **I like it** |
| --- | --- | --- | --- | --- | --- |
| If a health app provides one-click emergency help and automatically sends location information to family members, village doctors, or nearby medical institutions, how would you feel? | ○ | ○ | ○ | ○ | ○ |
| If a health app does not provide one-click emergency help or location-sending functions, how would you feel? | ○ | ○ | ○ | ○ | ○ |

**14. Human customer service support**

| **Item** | **I dislike it** | **I can tolerate it** | **Neutral** | **It should be that way** | **I like it** |
| --- | --- | --- | --- | --- | --- |
| If a health app provides telephone or online human customer service to help you solve app-use problems or health consultation issues, how would you feel? | ○ | ○ | ○ | ○ | ○ |
| If a health app does not provide human customer service support, how would you feel? | ○ | ○ | ○ | ○ | ○ |

**15. Medical consultation support**

| **Item** | **I dislike it** | **I can tolerate it** | **Neutral** | **It should be that way** | **I like it** |
| --- | --- | --- | --- | --- | --- |
| If a health app can connect local village doctors with remote specialists and support video consultations and online medical advice, how would you feel? | ○ | ○ | ○ | ○ | ○ |
| If a health app cannot provide medical consultation, video consultation, or online medical advice, how would you feel? | ○ | ○ | ○ | ○ | ○ |

**16. Medicine purchase and delivery**

| **Item** | **I dislike it** | **I can tolerate it** | **Neutral** | **It should be that way** | **I like it** |
| --- | --- | --- | --- | --- | --- |
| If a health app supports online medicine purchase from local pharmacies and provides home delivery services, how would you feel? | ○ | ○ | ○ | ○ | ○ |
| If a health app does not support online medicine purchase or home delivery services, how would you feel? | ○ | ○ | ○ | ○ | ○ |

**17. Medical policy inquiry**

| **Item** | **I dislike it** | **I can tolerate it** | **Neutral** | **It should be that way** | **I like it** |
| --- | --- | --- | --- | --- | --- |
| If a health app can help you quickly understand medical insurance benefits, reimbursement policies, and relevant medical policies, how would you feel? | ○ | ○ | ○ | ○ | ○ |
| If a health app does not provide inquiries about medical policies, medical insurance benefits, or reimbursement information, how would you feel? | ○ | ○ | ○ | ○ | ○ |

**18. Reminder function**

| **Item** | **I dislike it** | **I can tolerate it** | **Neutral** | **It should be that way** | **I like it** |
| --- | --- | --- | --- | --- | --- |
| If a health app provides medication reminders, medical visit reminders, physical examination reminders, and vaccination reminders, how would you feel? | ○ | ○ | ○ | ○ | ○ |
| If a health app does not provide reminders for medication, medical visits, physical examinations, or vaccinations, how would you feel? | ○ | ○ | ○ | ○ | ○ |

**19. Health monitoring and management**

| **Item** | **I dislike it** | **I can tolerate it** | **Neutral** | **It should be that way** | **I like it** |
| --- | --- | --- | --- | --- | --- |
| If a health app provides health monitoring and management functions such as step records, dietary advice, and sleep analysis, how would you feel? | ○ | ○ | ○ | ○ | ○ |
| If a health app does not provide health monitoring and management functions, how would you feel? | ○ | ○ | ○ | ○ | ○ |

**20. Infectious disease warning**

| **Item** | **I dislike it** | **I can tolerate it** | **Neutral** | **It should be that way** | **I like it** |
| --- | --- | --- | --- | --- | --- |
| If a health app combines local disease control information and timely pushes infectious disease warnings and protection guidelines, how would you feel? | ○ | ○ | ○ | ○ | ○ |
| If a health app does not provide infectious disease warnings or protection guidelines, how would you feel? | ○ | ○ | ○ | ○ | ○ |

**21. Weather forecast and health advice**

| **Item** | **I dislike it** | **I can tolerate it** | **Neutral** | **It should be that way** | **I like it** |
| --- | --- | --- | --- | --- | --- |
| If a health app provides real-time weather forecasts and gives health advice based on weather conditions, how would you feel? | ○ | ○ | ○ | ○ | ○ |
| If a health app does not provide weather forecasts or weather-related health advice, how would you feel? | ○ | ○ | ○ | ○ | ○ |

**22. Health device compatibility**

| **Item** | **I dislike it** | **I can tolerate it** | **Neutral** | **It should be that way** | **I like it** |
| --- | --- | --- | --- | --- | --- |
| If a health app can be compatible with common health monitoring devices such as blood pressure monitors, blood glucose meters, and smart bracelets, and synchronize health data, how would you feel? | ○ | ○ | ○ | ○ | ○ |
| If a health app cannot be compatible with common health monitoring devices or synchronize health data, how would you feel? | ○ | ○ | ○ | ○ | ○ |

**23. Family health sharing**

| **Item** | **I dislike it** | **I can tolerate it** | **Neutral** | **It should be that way** | **I like it** |
| --- | --- | --- | --- | --- | --- |
| If a health app allows your children or family members to remotely view your health data so that they can care for and assist you in time, how would you feel? | ○ | ○ | ○ | ○ | ○ |
| If a health app does not support remote viewing or sharing of health data with family members, how would you feel? | ○ | ○ | ○ | ○ | ○ |

**24. Privacy protection**

| **Item** | **I dislike it** | **I can tolerate it** | **Neutral** | **It should be that way** | **I like it** |
| --- | --- | --- | --- | --- | --- |
| If a health app provides simple and understandable privacy settings and protects the security of your health data and personal information, how would you feel? | ○ | ○ | ○ | ○ | ○ |
| If a health app does not provide clear privacy settings or cannot adequately protect personal information security, how would you feel? | ○ | ○ | ○ | ○ | ○ |

**25. Online older adult classes**

| **Item** | **I dislike it** | **I can tolerate it** | **Neutral** | **It should be that way** | **I like it** |
| --- | --- | --- | --- | --- | --- |
| If a health app teaches you how to use smart devices and common mobile phone functions through video tutorials or voice explanations, how would you feel? | ○ | ○ | ○ | ○ | ○ |
| If a health app does not provide teaching content about smart devices or mobile phone functions, how would you feel? | ○ | ○ | ○ | ○ | ○ |

**26. Entertainment module**

| **Item** | **I dislike it** | **I can tolerate it** | **Neutral** | **It should be that way** | **I like it** |
| --- | --- | --- | --- | --- | --- |
| If a health app provides entertainment content such as opera, music, crosstalk, and dance videos to enrich your daily life, how would you feel? | ○ | ○ | ○ | ○ | ○ |
| If a health app does not provide entertainment content, how would you feel? | ○ | ○ | ○ | ○ | ○ |

**27. Social interaction support**

| **Item** | **I dislike it** | **I can tolerate it** | **Neutral** | **It should be that way** | **I like it** |
| --- | --- | --- | --- | --- | --- |
| If a health app provides a communication platform for older adults to help you communicate with others and reduce loneliness, how would you feel? | ○ | ○ | ○ | ○ | ○ |
| If a health app does not provide social interaction or a communication platform, how would you feel? | ○ | ○ | ○ | ○ | ○ |

**28. Health knowledge popularization**

| **Item** | **I dislike it** | **I can tolerate it** | **Neutral** | **It should be that way** | **I like it** |
| --- | --- | --- | --- | --- | --- |
| If a health app disseminates health maintenance and disease prevention knowledge through videos, text, audio, and other forms, how would you feel? | ○ | ○ | ○ | ○ | ○ |
| If a health app does not provide health knowledge popularization content, how would you feel? | ○ | ○ | ○ | ○ | ○ |
